# An EGFR T790M-mutated lung adenocarcinoma undergoing large-cell neuroendocrine carcinoma transformation after osimertinib therapy: a case report

**DOI:** 10.1186/s13256-020-02447-0

**Published:** 2020-08-07

**Authors:** Shinichi Miyazaki, Yasumasa Kuno, Shunsaku Hayai, Ryo Teramachi, Ryo Yamashita, Yusuke Saito, Kosuke Higuchi, Yoshiharu Nara, Takuya Ikeda

**Affiliations:** 1 Department of Respiratory Medicine, Yokkaichi Municipal Hospital, 2-2-37, Shibata, Yokkaichi-shi, Mie 510-0822 Japan; 2 Department of Respiratory Medicine, Nagoya University Graduate School of Medicine, 65 Tsurumai-cho, Showa-ku, Nagoya, 466-8560 Japan; 3 Department of Pharmacy, Yokkaichi Municipal Hospital, 2-2-37, Shibata, Yokkaichi-shi, Mie 510-0822 Japan; 4 Department of Pathology, Yokkaichi Municipal Hospital, 2-2-37, Shibata, Yokkaichi-shi, Mie 510-0822 Japan

**Keywords:** Epidermal growth factor receptor, T790M, Osimertinib, Large-cell neuroendocrine carcinoma, Transformation

## Abstract

**Background:**

Osimertinib, a third-generation epidermal growth factor receptor tyrosine kinase inhibitor, is selective for both epidermal growth factor receptor tyrosine kinase inhibitor–sensitizing and T790M resistance mutations. Almost all patients who initially respond to an epidermal growth factor receptor tyrosine kinase inhibitor subsequently report disease progression. Epidermal growth factor receptor–dependent resistance mechanisms, bypass pathway activation, and histological transformation have been reported with osimertinib therapy.

**Case presentation:**

We report a case of a 64-year-old Asian man with epidermal growth factor receptor T790M-positive adenocarcinoma that transformed to epidermal growth factor receptor T790M-negative large-cell neuroendocrine carcinoma after osimertinib therapy. A prompt rebiopsy revealed a rare mechanism of resistance to epidermal growth factor receptor tyrosine kinase inhibitor, and subsequently treatment with carboplatin and etoposide was effective.

**Conclusions:**

Despite the promising emergence of circulating tumoral DNA testing, this case report emphasizes the importance of rebiopsy of a progressive epidermal growth factor receptor–mutant tumor.

## Background

Lung cancer is the most common cause of cancer mortality worldwide. Non-small-cell lung cancer (NSCLC) accounts for 85% of cases and includes adenocarcinoma, squamous cell carcinoma, and large-cell carcinoma. The epidermal growth factor receptor (EGFR) gene is one of the most important oncogenes in NSCLC. Mutations in EGFR tyrosine kinase are observed in 51.4% of advanced NSCLC adenocarcinoma cases in Asian populations compared, with approximately 20% among the white population [1]. EGFR mutations are more common in nonsmokers and women. Osimertinib therapy is the first-line treatment for patients with metastatic NSCLC whose tumors have EGFR exon 19 deletions or exon 21 L858R mutations. It is also effective against tumors that harbor the T790M EGFR mutation responsible for 50% of acquired resistance to first- and second-generation EGFR tyrosine kinase inhibitors (TKIs) [2]. Almost all patients who initially respond to an EGFR TKI subsequently report disease progression. EGFR-dependent resistance mechanisms (such as the C797S point mutation), bypass pathway activation (such as mesenchymal–epithelial transition factor amplification), and histological transformation (such as small cell carcinoma) have been reported with osimertinib therapy [3]. We report a rare case of a patient in whom EGFR T790M-positive adenocarcinoma transformed into large-cell neuroendocrine carcinoma (LCNEC) after osimertinib therapy.

## Case presentation

**Fig. 1:**
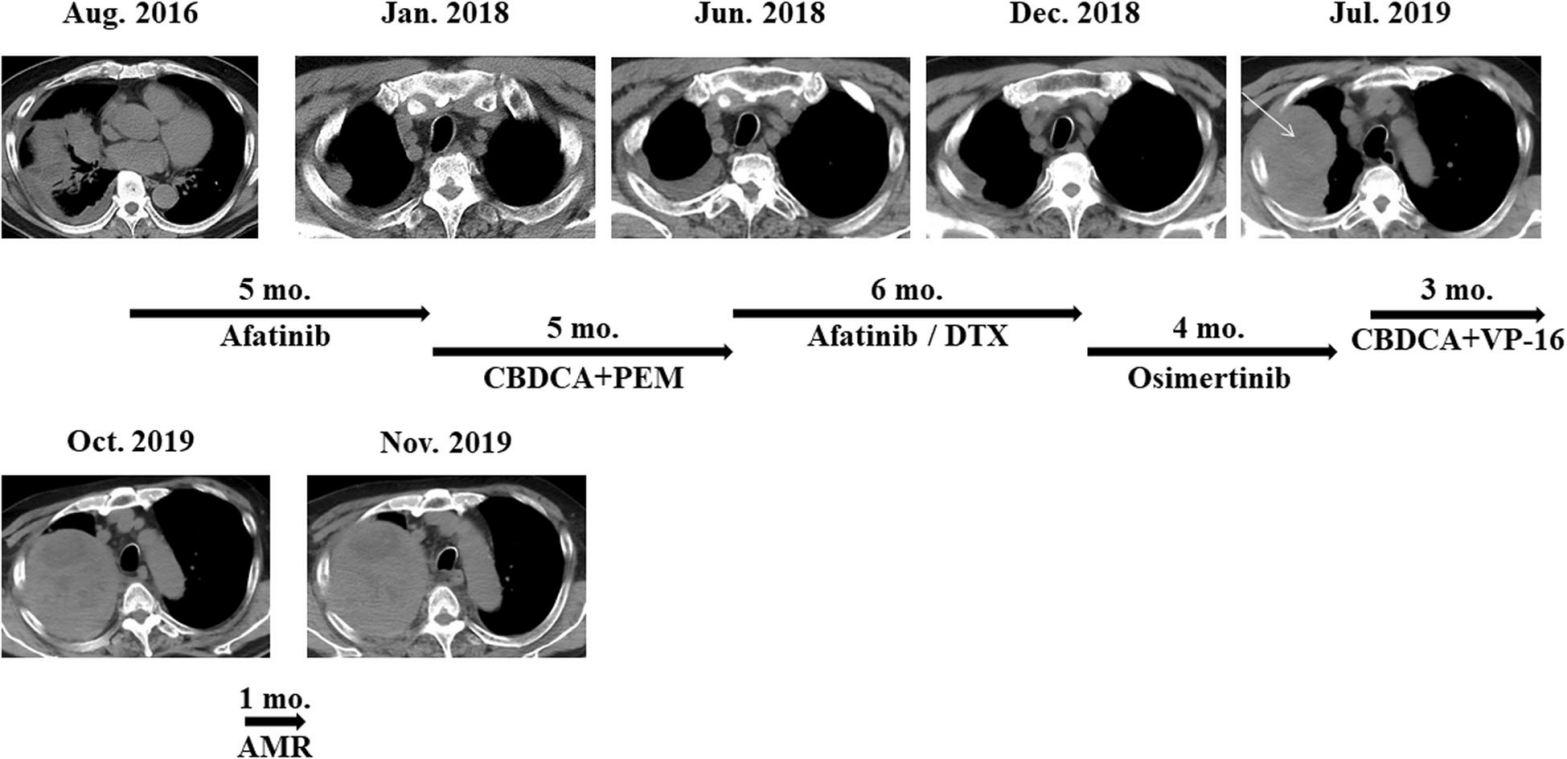
Clinical history of the patient. A rebiopsy was performed from the right pleural metastasis (*white arrow*)

**Fig. 2:**
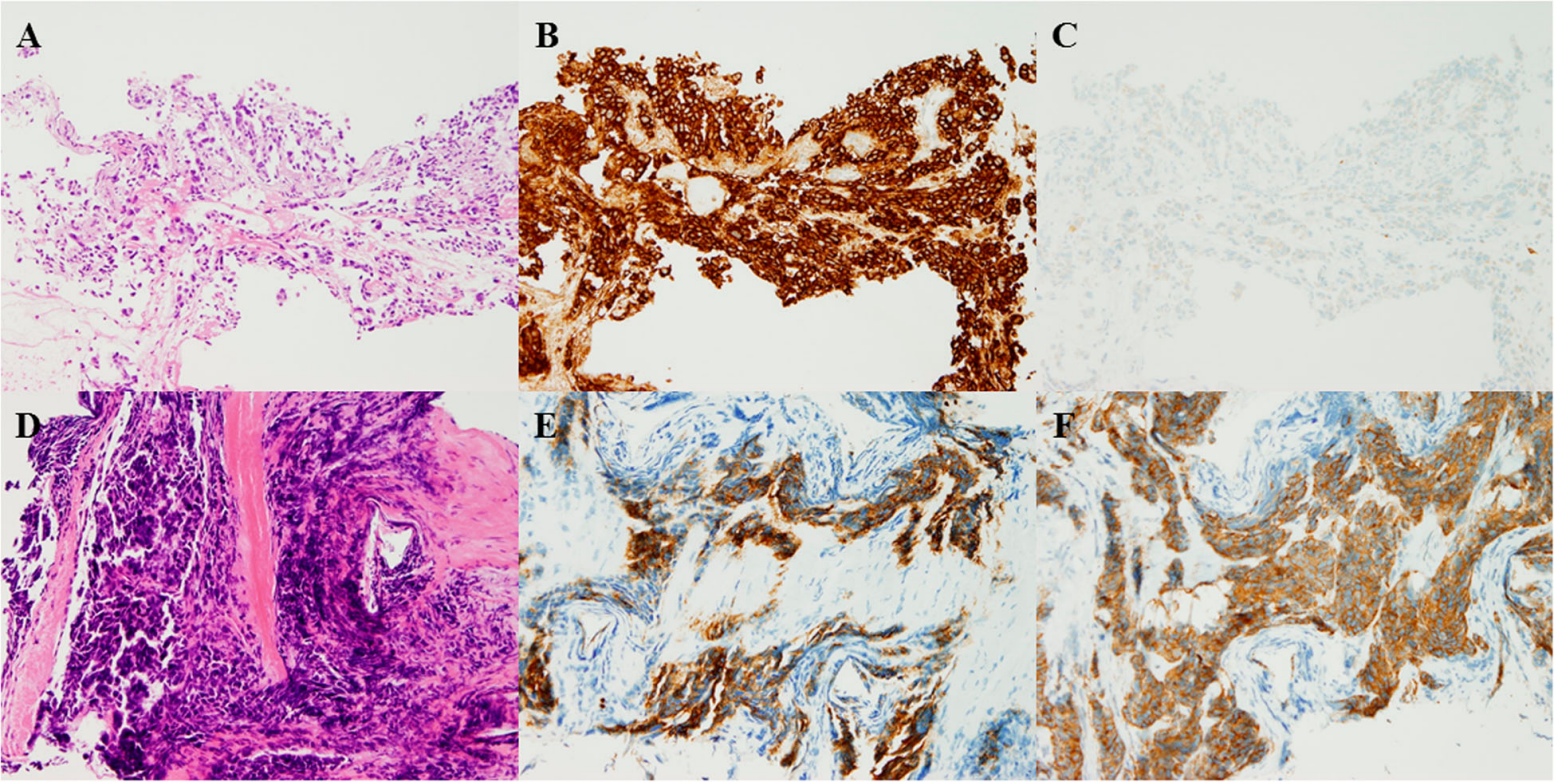
**a**–**c** Histopathological results of the biopsy specimen. **a** Hematoxylin and eosin staining. **b** Carcinoembryonic antigen staining (CEA). **c** Synaptophysin staining. Original magnification, × 100. **d**–**f** Histology of pleural biopsy specimen. **d** Hematoxylin and eosin staining. **e** CEA staining. **f** synaptophysin staining. Original magnification, × 100

A 64-year-old Asian man with a 46–pack-year smoking history was referred to our department for progressive exertional dyspnea over the course of 1 week. An extensive workup demonstrated a right middle lobe mass, right hilar lymphadenopathy, bilateral pulmonary nodules, and right pleural effusion with pleural nodules, as well as nodules in the peritoneum, mesentery, and omentum (Fig. 1). Histopathological and molecular analyses of transbronchial biopsy specimens from the right middle lobe revealed adenocarcinoma; the malignant epithelial cells were positive for carcinoembryonic antigen (CEA) and negative for synaptophysin (Fig. 2), and they harbored an exon 19 deletion mutation in EGFR. The patient was initially treated with afatinib, which resulted in a partial response.

Seventeen months later, computed tomography (CT) revealed progression of the primary lesion in the right middle lobe and new metastasis to the right pleura. Five cycles of carboplatin (CBDCA) and pemetrexed (PEM) were administered as second-line treatment, which subsequently stabilized the disease. Although one cycle of maintenance therapy with PEM was administered, the lesions of the right middle lobe and pleura progressed. Over the next 7 months, neither six cycles of docetaxel treatment nor afatinib rechallenge was effective. A liquid biopsy revealed EGFR-T790M mutation, and the patient received osimertinib therapy. Although this maintained the stable disease status for 4 months, rapid progression of the right pleural metastasis occurred subsequently. A CT-guided biopsy of the pleura was performed, and LCNEC characterized by positive CEA and synaptophysin staining was identified by histologic examination (Fig. 2). Molecular analysis revealed the EGFR exon 19 deletion without T790M mutation. Thus, a combination of CBDCA and etoposide (VP-16) was initiated, and the patient had stable disease for 3 months. After three cycles of CBDCA and VP-16, the right pleural metastasis progressed, and the treatment was changed to amrubicin (AMR). After one cycle of AMR, he developed superior vena cava syndrome, and he died 41 months after the initial presentation.

## Discussion

We report a case of a patient with EGFR T790M-positive adenocarcinoma that transformed to EGFR T790M-negative LCNEC after osimertinib therapy. EGFR exon 19 deletion was identified in both the adenocarcinoma and LCNEC. To the best of our knowledge, this is the fourth description of adenocarcinoma transformation to LCNEC after osimertinib therapy [4–6]. One recent study investigating the mechanisms of resistance to osimertinib in 73 consecutive patients reported only small cell lung cancer (SCLC) transformation in 4 patients [7]. LCNEC shares several clinical characteristics and genomic features with SCLC [8], and, similarly to cases of adenocarcinoma transforming to SCLC [9], the underlying mechanism leading to neuroendocrine differentiation may be the inactivation of retinoblastoma 1 (RB1) and tumor protein 53 (TP53). Inactivation of RB1 and TP53 was observed in pretransformed and post-transformed EGFR-mutant lung adenocarcinoma [9]. However, RB1 loss alone is not sufficient to drive SCLC transformation [10], indicating that other changes must occur in tumors to promote SCLC transformation. The preferential APOBEC (apolipoprotein B mRNA editing enzyme catalytic polypeptide-like)-induced hypermutation was observed in transformed SCLCs, whereas EGFR T790M-positive lung adenocarcinoma had rare APOBEC-associated mutations. The APOBEC mutational process may induce SCLC transformation through genomic instability.

Several demographic and pathological factors are associated with EGFR mutation prevalence. EGFR mutations tend to be more common among patients with an adenocarcinoma histology and among nonsmokers [11]. Among Asian populations, the average incidences of EGFR mutations were 31% overall, 47% among patients with adenocarcinoma, and 56% among nonsmokers. Among white populations, the average incidences of EGFR mutations were 7% overall, 13% among patients with adenocarcinoma, and 35% among nonsmokers. The reason for the high frequency of EGFR mutations in Asian patients is unclear, and genome-wide association studies may identify loci that are specifically related to EGFR-targeted carcinogenesis [12]. A multicenter phase II study on cisplatin-etoposide chemotherapy for LCNEC demonstrated that the major efficacy endpoints were similar to those of SCLC [13], and researchers have recommended platinum-etoposide chemotherapy treatment [14]. In 2018, Horn *et al.* reported that the addition of atezolizumab to chemotherapy in the first-line treatment of extensive stage SCLC increased overall survival and progression-free survival significantly compared with chemotherapy alone [15]. These data suggest that adding an immune checkpoint inhibitor to etoposide-platinum in the front-line setting improves prognosis for patients with LCNEC. At the time we treated our patient, however, addition of atezolizumab to chemotherapy was not approved, owing to the restrictions of the national health insurance system in Japan.

**Fig. 3:**
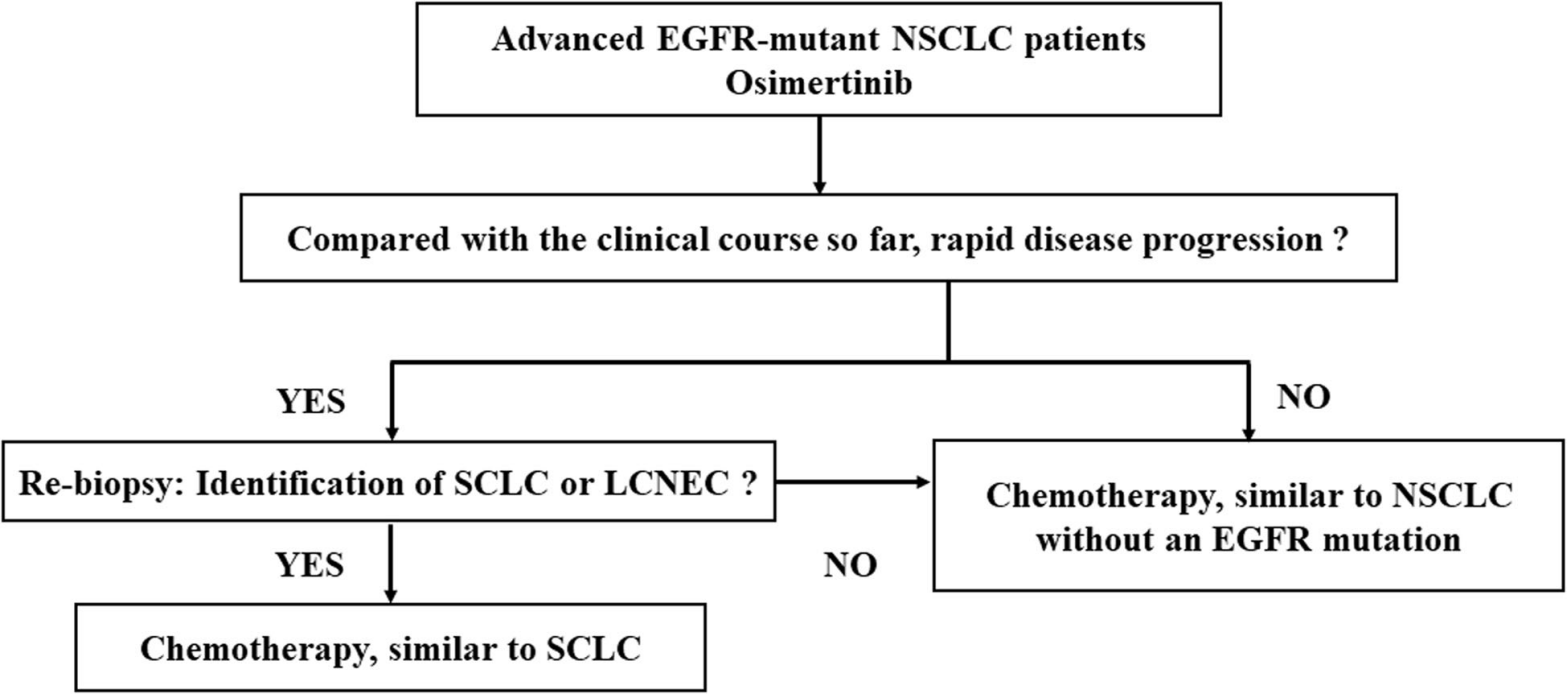
Treatment algorism for advanced epidermal growth factor receptor–mutant patients with non-small-cell lung cancer

Although rebiopsy has a pivotal role in investigating mechanisms of resistance to EGFR TKIs, it is not always feasible. Previous studies have reported that the rate of rebiopsy is approximately 50% [16, 17]; the reasons for not performing a rebiopsy were inaccessible tumors (5%), deterioration in performance status (4%), and patient refusal (22%) [18]. In the present study, rebiopsy revealed that transformation to LCNEC was the mechanism underlying the resistance to EGFR TKI and subsequently led to effective treatment of our patient (Fig. 3).

## Conclusions

We report a case of a patient in whom EGFR T790M-positive adenocarcinoma transformed into LCNEC after osimertinib therapy. Rebiopsy of the tumor after disease progression was instrumental in determining the mechanism of osimertinib resistance and led to effective treatment.

## Data Availability

Data are available from the corresponding author on reasonable request.

## Abbreviations

AMR: Amrubicin
APOBEC: Apolipoprotein B mRNA editing enzyme catalytic polypeptide-like
CBDCA: Carboplatin
CEA: Carcinoembryonic antigen
CT: Computed tomography
EGFR: Epidermal growth factor receptor
LCNEC: Large-cell neuroendocrine carcinoma
NSCLC: Non-small-cell lung cancer
PEM: Pemetrexed
RB1: Retinoblastoma 1
SCLC: Small cell lung cancer
TKI: Tyrosine kinase inhibitor
TP53: Tumor protein 53
VP-16: Etoposide
